# Alterations in gut microbiota and inflammatory cytokines after administration of antibiotics in mice

**DOI:** 10.1128/spectrum.03095-23

**Published:** 2024-06-20

**Authors:** Wang Gao, Xingyu Liu, Shuobo Zhang, Jingxia Wang, Bo Qiu, Junhua Shao, Weixin Huang, Yilun Huang, Mingfei Yao, Ling-Ling Tang

**Affiliations:** 1 Jinan Microecological Biomedicine Shandong Laboratory, Jinan, China; 2 State Key Laboratory for Diagnosis and Treatment of Infectious Diseases, National Clinical Research Center for Infectious Diseases, Collaborative Innovation Center for Diagnosis and Treatment of Infectious Diseases, The First Affiliated Hospital, School of Medicine, Zhejiang University, Hangzhou, China; 3 Shandong First Medical University and Shandong Academy of Medical Sciences, Jinan, China; 4 Shulan (Hangzhou) Hospital Affiliated to Zhejiang Shuren University, Shulan International Medical College, Hangzhou, China; 5 Shaoxing Tongchuang Biotechnology Co., Ltd, Shaoxing, China; 6 Alberta Institute, Wenzhou Medical University, Wenzhou, China; Huazhong University of Science and Technology, Wuhan, China

**Keywords:** antibiotic, gut microbiota, cytokine, inflammatory response, dysbiosis

## Abstract

Antibiotics are widely used to treat bacterial infection and reduce the mortality rate, while antibiotic overuse can cause gut microbiota dysbiosis. The impact of antibiotics on gut microbiota is not fully understood. In our study, four commonly used antibiotics (ceftazidime, cefoperazone-sulbactam, imipenem-cilastatin, and moxifloxacin) were given subcutaneously to mice, and their impacts on the gut microbiota composition and serum cytokine levels were evaluated through 16S rRNA analysis and a multiplex immunoassay. Antibiotic treatment markedly reduced gut microbiota diversity and changed gut microbiota composition. Antibiotic treatment significantly increased and decreased the abundance of Firmicutes and Bacteroidota, respectively. The antibiotic treatments increased the abundance of opportunistic pathogens such as *Enterococcus* and decreased that of *Lachnospiraceae* and *Muribaculaceae*. For moxifloxacin, the significantly high abundance of *Enterococcus* and *Klebsiella* was observed after 14 and 21 days of treatment. However, a relatively low abundance of opportunistic pathogens was found after 14 days of imipenem-cilastatin treatment. Additionally, the serum levels of various pro-inflammatory cytokines, such as IL-1β, IL-12 (p70), and IL-17, significantly increased after 21 days of antibiotic treatments. Overall, these results provide a guide for rational use of antibiotics in clinical settings: short-term use of moxifloxacin is recommended with regard to gut microbiota health, and the 14-day use of imipenem-cilastatin may have a less severe impact than other antibiotics.

**IMPORTANCE:**

Antibiotic treatments are directly associated with changes in gut microbiota and are effective against both pathogens and beneficial bacteria. Gut microbiota dysbiosis induced by antibiotic treatment could increase the risk of some diseases. Therefore, an adequate understanding of gut microbiota changes after antibiotic use is crucial. In this study, we investigated the effects of continuous treatment with antibiotics on gut microbiota, serum cytokines, and intestinal inflammatory response. Our results suggest that short-term use of moxifloxacin is recommended, and the 14-day use of imipenem-cilastatin may have a less severe effect on gut microbiota health than cefoperazone-sulbactam. These results provide useful guidance on the rational use of antibiotics with regard to gut microbiota health.

## INTRODUCTION

Antibiotics were first discovered in the 1920s by the British scientist Fleming, but it was not until the outbreak of World War II that penicillin was accepted and used as a key drug for the treatment of infectious diseases. In the late 1940s, the American scientist Waksman discovered streptomycin, a potent antibiotic that was particularly effective against tuberculosis (1, 2). Subsequently, scientists discovered many other antibiotics and applied them on a large scale in industrial production, propelling rapid advancements in the medical field (3). However, there is a growing concern about the overuse of antibiotics in clinical settings. Many intestinal and extra-intestinal illnesses have been related to antibiotic overuse, including antibiotic-associated gut mucosal injury, inflammatory bowel disease, colon cancer, asthma, and diabetes (4, 5). A meta-analysis found that antibiotic therapy significantly increased the risk of type 2 diabetes in individuals over the age of 50 (6).

Gut microbiota plays an important role in maintaining human health (7, 8). It positively affects the immune and metabolic responses of the body (9) and helps the immune system in developing tolerance to autoantigens and avoiding the development of autoimmune diseases (10, 11). Gut microbiota also significantly affects neurocognitive and mental health (12, 13). Although antibiotic treatment is effective in controlling infection, the use of antibiotics may directly affect the gut microbiota. The overuse of antibiotics may lead to gut microbiota dysbiosis and increase the likelihood of *Clostridioides difficile* infection (14, 15). Zwittink et al. found that treatment with ceftazidime (CAZ) decreased the relative abundance of *Escherichia coli-Shigella* and *Streptococcus*, ultimately affecting the gut microbiota composition in infants (16). Another study showed that long-term cefoperazone-sulbactam (CPZ_SAM) treatment affected the function of the coagulation system (17). Burdet et al. found that treatment with moxifloxacin (MOX) reduced bacterial diversity in a concentration-dependent manner (18). Additionally, there is a significant correlation between gut microbiota dysbiosis, inflammatory cytokines changes, and inflammatory disorders (19). Gut microbiota dysbiosis can lead to increased intestinal permeability by damaging the epithelial cells and intercellular junctions of the intestinal barrier (20). This facilitates the migration of intestinal pathogens and harmful substances like endotoxins into the bloodstream, causing local or systemic inflammation (21). Dysbiosis of gut microbiota can constantly activate M1 macrophages, resulting in the development of chronic inflammatory and autoimmune disorders (22). Therefore, it is crucial to understand the effect of antibiotics on gut microbiota and inflammatory response. However, dynamic alterations in gut microbiota caused by continuous antibiotic treatment have not been systematically studied, and few studies have compared the gut microbiota composition after various antibiotic treatments.

Gram-negative bacteria are known to be pathogenic, and three types of antibiotics are widely used to treat gram-negative bacterial infections in clinical settings, including third-generation cephalosporins, quinolones, and carbapenems. Due to the presence of multidrug resistance genes, extended-spectrum β-lactamase (ESBL)-producing gram-negative bacteria are resistant to both β-lactam and quinolone antibiotics such as ceftazidime and moxifloxacin (23, 24). Such antibiotics are widely used to treat infections caused by non-ESBL-producing gram-negative bacteria. β-lactam/β-lactamase inhibitor combinations, such as cefoperazone-sulbactam and imipenem-cilastatin (IPM_CS), have increasingly become central in the treatment of serious infections caused by ESBL-producing gram-negative bacteria (25–28).

In the present study, ceftazidime, moxifloxacin, cefoperazone-sulbactam, and imipenem-cilastatin were used to investigate the effect of antibiotics on gut microbiota. We aimed to investigate the dynamic effects of continuous treatment with widely used antibiotics on gut microbiota, serum cytokines, and intestinal inflammatory response and compare the differences among treatment groups to provide guidance on the rational use of antibiotics in clinical settings.

## RESULTS

### Changes in mice body weight and appearance of fecal matter during antibiotic treatment

**Fig 1:**
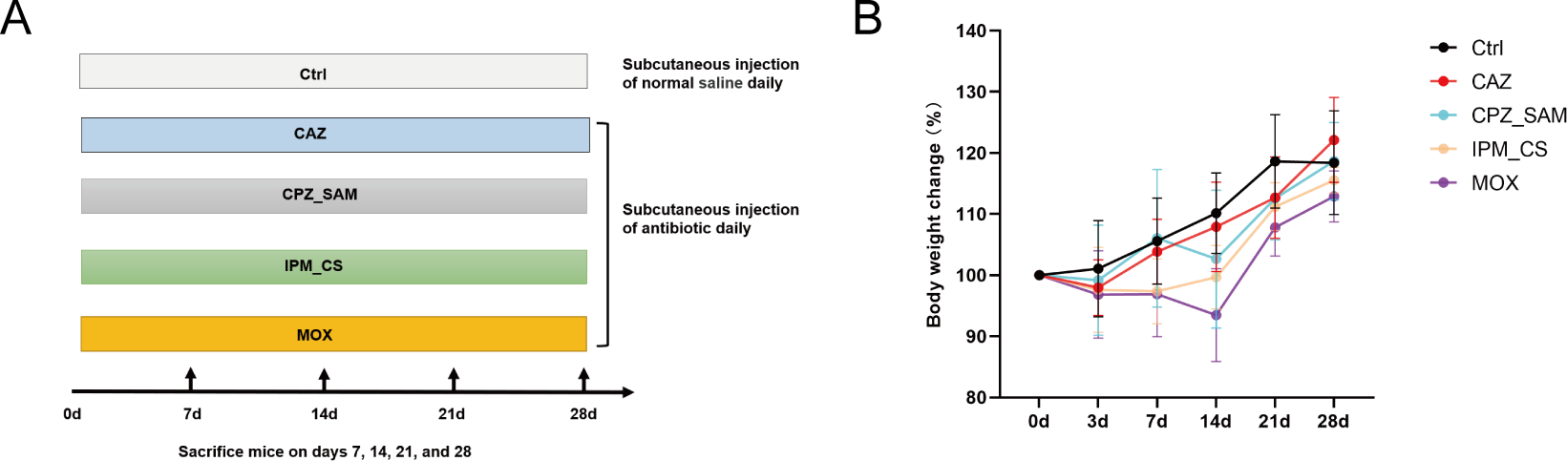
(**A**) Flow chart of the research design. BALB/c mice in antibiotic treatment groups were separately treated with imipenem-cilastatin, cefoperazone-sulbactam, ceftazidime, and moxifloxacin hydrochloride for 4 weeks. On days 7, 14, 21, and 28 of continuous antibiotic therapy, seven mice were sacrificed at each timepoint in the same antibiotic treatment group in order to collect serum, feces, and colon tissues. (**B**) Effect of antibiotic treatment on mice body weight over time. Abbreviations: IPM_CS, imipenem-cilastatin; CPZ_SAM, cefoperazone-sulbactam; CAZ, ceftazidime; MOX, moxifloxacin hydrochloride; and Ctrl, control.

Mice in the four antibiotic groups gained weight more slowly than control group mice (Fig. 1B). Mice in IPM_CS and MOX groups lost weight during the first 7 days of the antibiotic treatment period. The MOX group displayed the strongest suppression of body weight growth in comparison with other antibiotic groups. Additionally, fecal matter was analyzed; fecal matter showed a trend of softening from days 7 to 21 of antibiotic treatment, but normal consistency was observed after 21 days (Table S1).

### Effect of antibiotic treatment on diversity of gut microbiota

**Fig 2:**
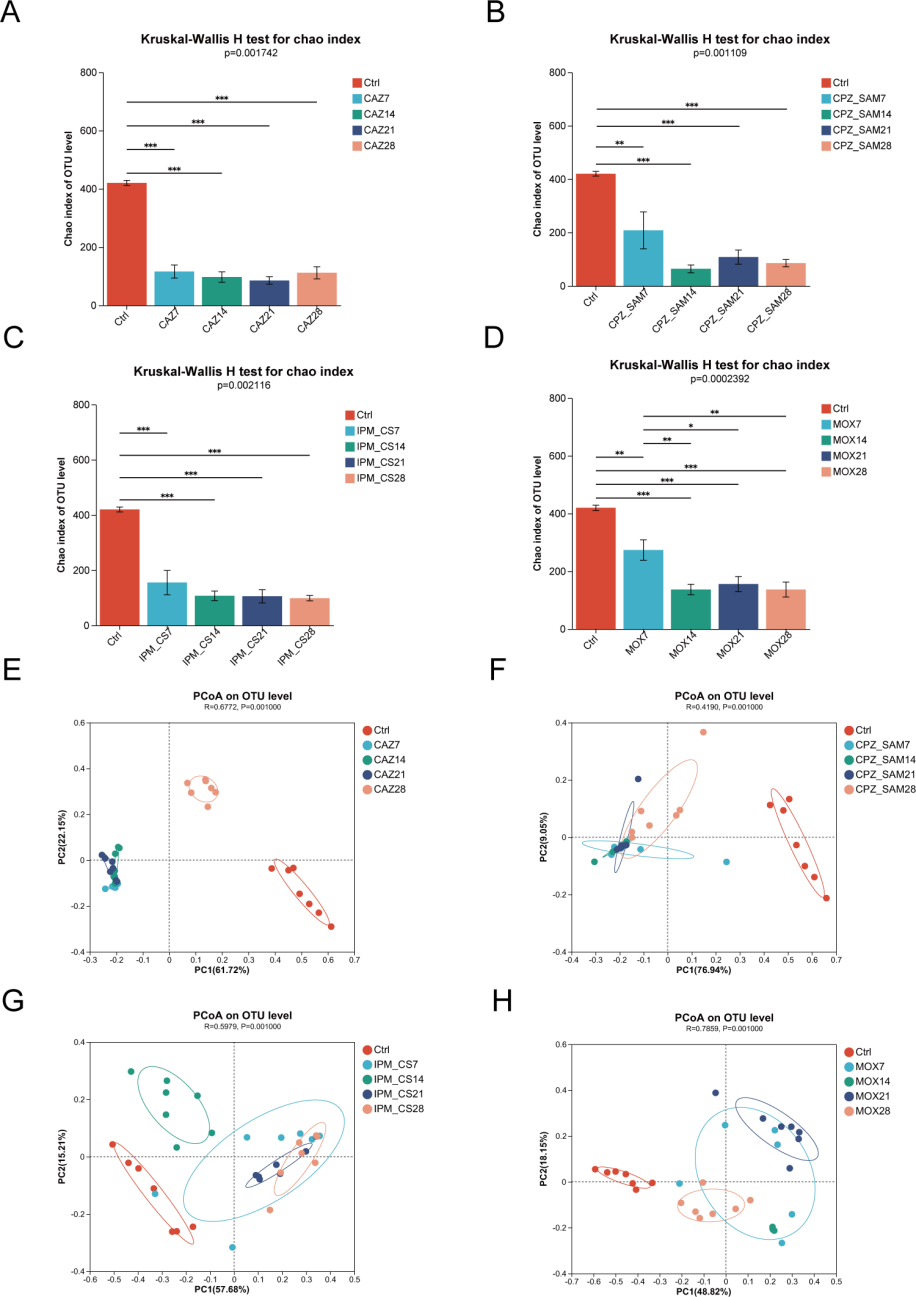
(A–D) Comparison of Chao index between the control group and different antibiotic treatment groups, indicating that antibiotic treatment altered the alpha diversity; *P < 0.05 and **P < 0.01. (E–H) Beta diversity was evaluated by performing principal coordinate analysis (PCoA) of weighted UniFrac distance. Abbreviations: IPM_CS, imipenem-cilastatin; CPZ_SAM, cefoperazone-sulbactam; CAZ, ceftazidime; MOX, moxifloxacin hydrochloride; Ctrl, control.

By calculating the Chao index, we assessed the species diversity and evenness of gut microbiota. On days 7, 14, 21, and 28, the Chao index was significantly lower in the CAZ (*P* < 0.001), CPZ_SAM (*P* < 0.01), IPM_CS (*P* < 0.001), and MOX groups (*P* < 0.01) than in the Ctrl group (Fig. 2A through D). Similarly, we found that the Shannon index on days 7, 14, 21, and 28 decreased significantly in these antibiotic treatment groups than in the Ctrl group (*P* < 0.001; Fig. S1). These findings indicate that all four antibiotic groups showed a significant reduction in the gut microbiota alpha diversity after antibiotic treatment. However, in the MOX group, the gut microbiota alpha diversity was significantly higher on day 7 than on days 14 (*P* < 0.01) and 21 (*P* < 0.05; Fig. 2D; Fig. S1).

We used principal coordinate analysis (PCoA) to compare and assess the differences in gut microbiota composition at different timepoints during antibiotic treatment. Significant differences in composition structure were observed among the control group and antibiotic treatment groups (Fig. 2E through H). Notably, the clustering in CAZ and CPZ_SAM groups on days 7, 14, and 21 indicated that the CAZ-treated and the CPZ_SAM-treated mice had similar gut microbiota composition on days 7, 14, and 21 (Fig. 2E and F, respectively). However, the gut microbiota compositions were changing with time when constantly administrated with IPM_CS or MOX (Fig. 2G and H).

### Effects of continuous antibiotic treatment on gut microbiota composition and abundance

**Fig 3:**
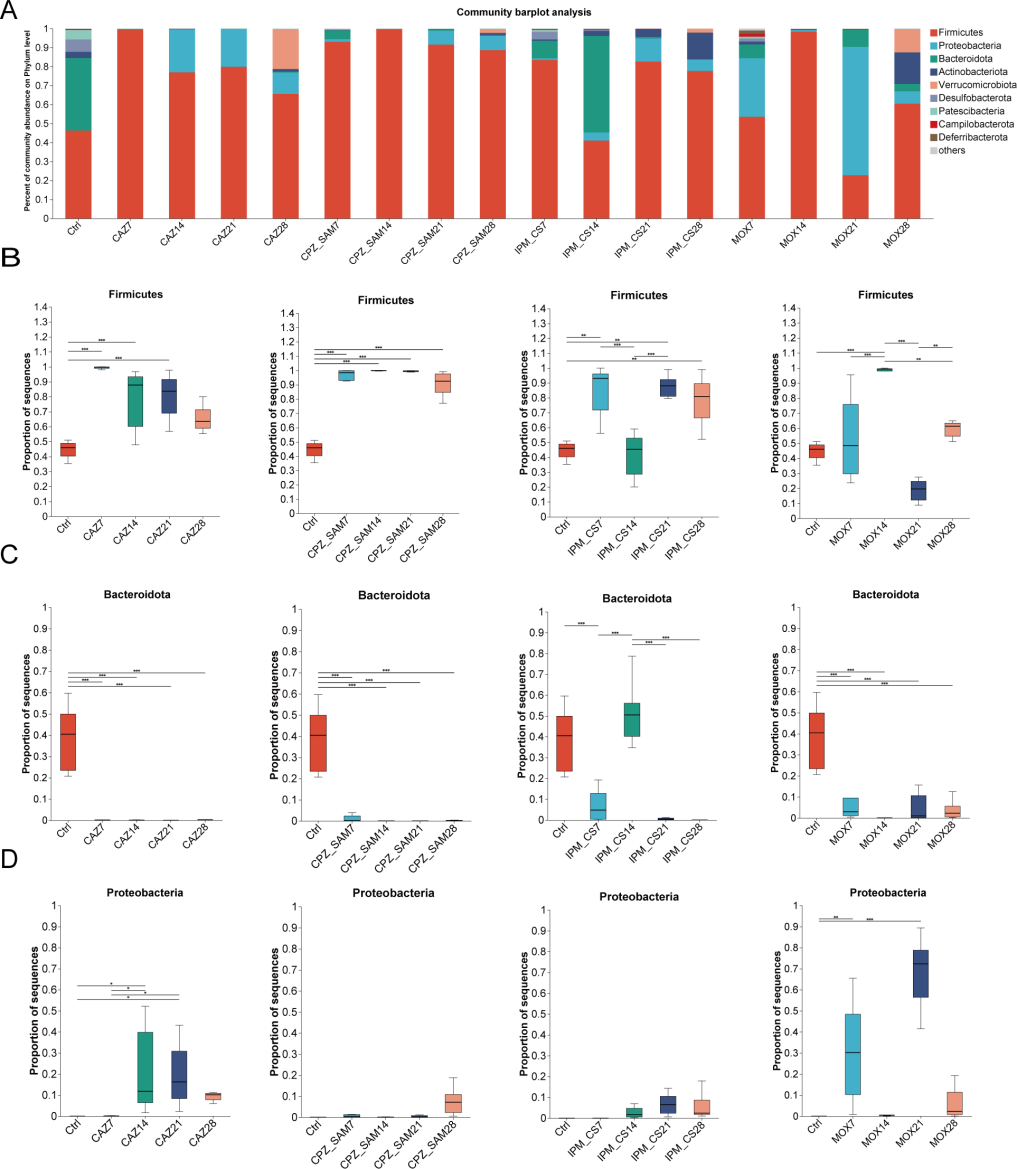
(**A**) Changes in microbial community composition at the phylum level in the four antibiotic treatment groups. Significant differences were observed in the average relative proportion of a phylum on different days within the same antibiotic treatment group: (**B**) Firmicutes, (**C**) Bacteroidota, and (**D**) Proteobacteria. Statistical analysis was performed using the Kruskal-Wallis rank sum test. Significant differences are marked with * (*P* < 0.05), ** (*P* < 0.01), and *** (*P* < 0.001). Abbreviations: IPM_CS, imipenem-cilastatin; CPZ_SAM, cefoperazone-sulbactam; CAZ, ceftazidime; MOX, moxifloxacin hydrochloride; Ctrl, control.

The composition of gut microbiota was evaluated in this section. Antibiotic treatments significantly altered gut microbiota composition and abundance; within a treatment group, the abundance of gut microbiota varied among different timepoints (Fig. 3A). The phyla with the most significant changes in relative abundance were Firmicutes, Bacteroidota, and Proteobacteria during antibiotic treatment. Generally, the abundance of Firmicutes was increased significantly, while Bacteroidota was decreased after continuous ingestion of antibiotics (Fig. 3B and C). In IPM_CS group, the abundance of Firmicutes was high on days 7, 21, and 28, but there was a decrease on day 14 when the abundance of Bacteroidota was recovered. Figure 3D showed that there was no significant change in the abundance of Proteobacteria in IPM_CS and CPZ_SAM groups. However, the abundance of Proteobacteria (*P* < 0.05) was significantly higher in the CAZ group than in the Ctrl group on days 14 and 21. In MOX group, Proteobacteria was increased significantly on days 7 (*P* < 0.01) and 21 (*P* < 0.001) but extremely low at other timepoints.

**Fig 4:**
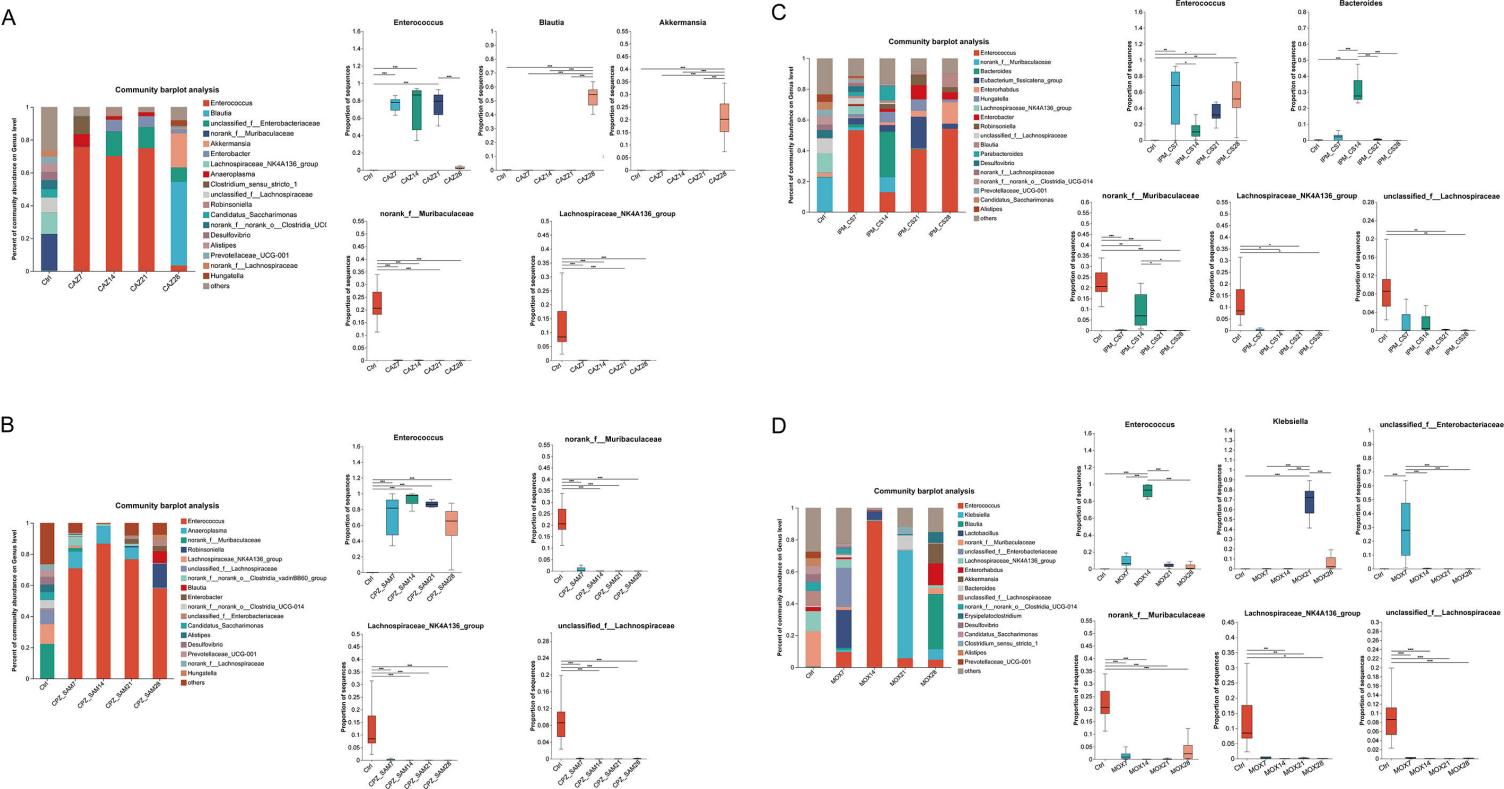
Mean relative abundance of gut microbiota at the genus level determined on the basis of 16S rRNA gene sequencing of mouse fecal samples. Significant differences were observed in the mean relative abundance of a genus on different days within the same antibiotic treatment group: (**A**) CAZ group, (**B**) CPZ_SAM group, (**C**) IPM_CS group, and (**D**) MOX group. Statistical analysis was performed using the Kruskal-Wallis rank sum test. Significant differences are marked with * (*P* < 0.05), **(*P* < 0.01), and *** (*P* < 0.001). Abbreviations: IPM_CS, imipenem-cilastatin; CPZ_SAM, cefoperazone-sulbactam; CAZ, ceftazidime; MOX, moxifloxacin hydrochloride; Ctrl, control.

Antibiotic treatments significantly affected the gut microbiota composition and abundance at the genus level (Fig. 4). The abundance of *Enterococcus* was extremely low in the Ctrl group. However, antibiotic treatment significantly increased the abundance of *Enterococcus*. Compared to the Ctrl group, the CAZ group showed a significantly higher abundance of *Enterococcus* (*P* < 0.001) on days 7, 14, and 21 than the Ctrl group. Notably, the abundance of *Enterococcus* (*P* < 0.001) was significantly lower on day 28. The abundance of *Blautia* and *Akkermansia* was extremely low in the Ctrl group and on days 7, 14, and 21 in the CAZ treatment group, but their abundance was significantly increased on day 28 in the CAZ group (*P* < 0.001; Fig. 4A). In the CPZ_SAM group, the abundance of *Enterococcus* (*P* < 0.001) was significantly higher than in the Ctrl group, and *Enterococcus* became the dominant bacterial genus in this group (Fig. 4B).

The IPM_CS treatment group showed a significantly higher abundance of *Enterococcus* (*P* < 0.05) on days 7, 21, and 28 than the Ctrl group; however, the abundance of *Enterococcus* in the IPM_CS group was significantly lower on day 14 than on day 7 (*P* < 0.05). *Bacteroides* (*P* < 0.001) was abundant in the IPM_CS group on day 14, but its abundance was extremely low at other timepoints. Meanwhile, the abundance of *norank_f__Muribaculaceae* in the IPM_CS group was significantly higher on day 14 than on days 21 and 28 (*P* < 0.005; Fig. 4C).

The MOX group showed a higher abundance of *unclassified_f__Enterobacteriaceae* on day 7, higher abundance of *Enterococcus* (*P* < 0.001) on day 14, and higher abundance of *Klebsiella* (*P* < 0.001) on day 21 than on other days. Similar to the CAZ group, the MOX group showed a significantly higher abundance of *Blautia* (*P* < 0.001) on day 28 (Fig. 4D). Additionally, the abundance of *norank_f__Muribaculaceae*, *Lachnospiraceae_NK4A136_group*, and *unclassified_f__Lachnospiraceae* was significantly lower in all four antibiotic treatment groups than in the Ctrl group (Fig. 4).

### Comparative analysis of gut microbiota abundance between two antibiotic groups

**Fig 5:**
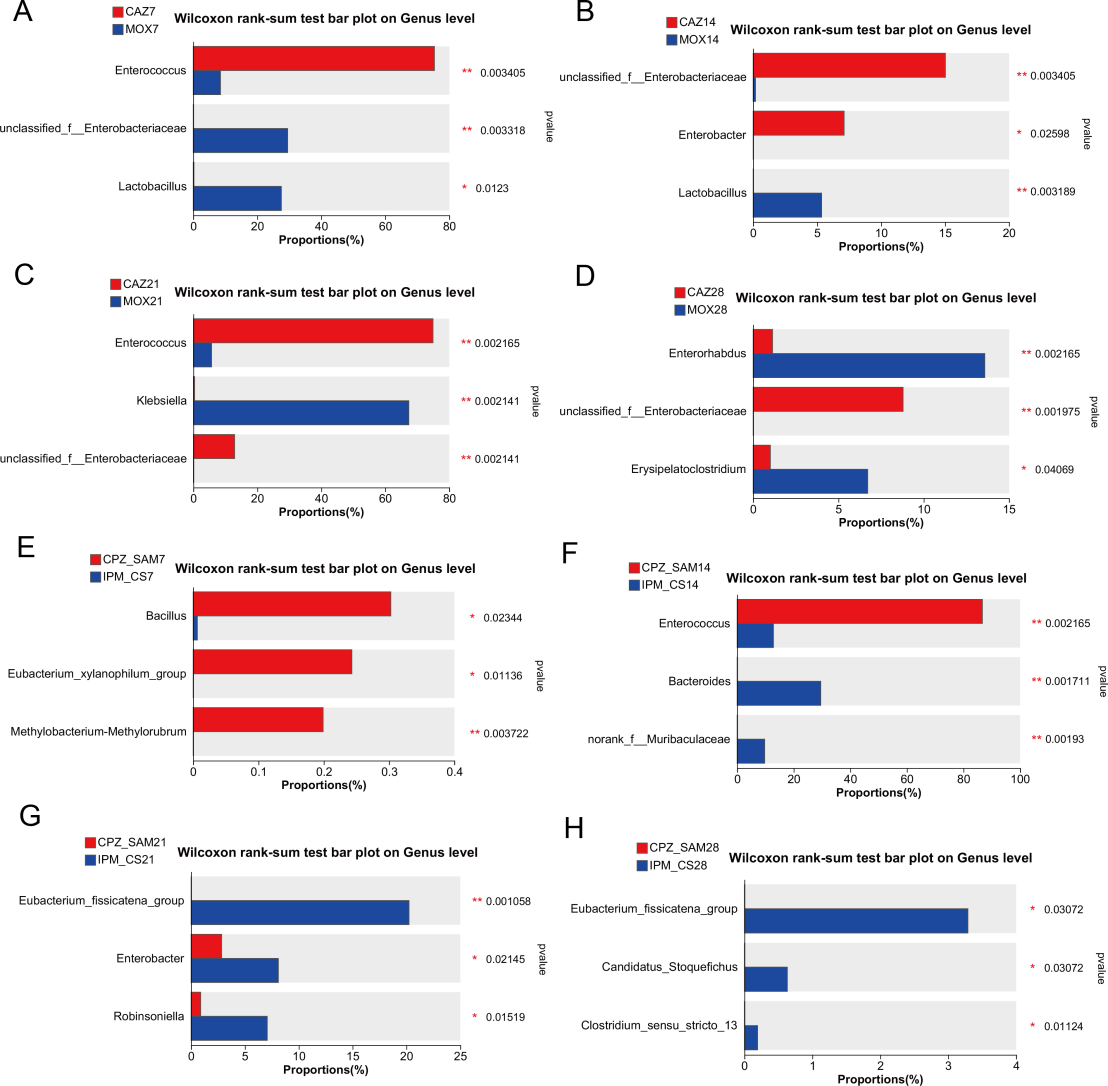
Proportions of top three genera with significant differences in average relative abundance between two antibiotic treatment groups. Statistical analysis was performed using the Wilcoxon rank-sum test. Significant differences are marked with * (*P* < 0.05), ** (*P* < 0.01), and *** (*P* < 0.001). Abbreviations: IPM_CS, imipenem-cilastatin; CPZ_SAM, cefoperazone-sulbactam; CAZ, ceftazidime; MOX, moxifloxacin hydrochloride; Ctrl, control.

CAZ and MOX are widely used for the treatment of infections caused by non-ESBL-producing gram-negative bacteria; in contrast, β-lactam/β-lactamase inhibitor combinations are effective against ESBL-producing gram-negative bacteria. We investigated the difference in gut microbiota abundance at the genus level between the CAZ and MOX groups and between the CPZ_SAM and IPM_CS groups (Fig. 5).

On day 7, the abundance of *Enterococcus* was significantly higher in the CAZ group than in the MOX group (*P* < 0.01). On day 7, the MOX group had a significantly higher abundance of *Unclassified_f__Enterobacteriaceae* and *Lactobacillus* than the CAZ group (Fig. 5A). On day 14, CAZ group had a significantly higher abundance of *unclassified_f__Enterobacteriaceae* (*P* < 0.01) and *Enterobacter* (*P* < 0.05) than the MOX group (Fig. 5B). A significantly higher abundance of *Enterococcus* in the CAZ group and *Klebsiella* in MOX group was observed after 21 days of antibiotic treatment (Fig. 5C).

On day 7, the CPZ_SAM group had a significantly higher abundance of *Bacillus* (*P* < 0.01) and *Eubacterium_xylanophilum_group* (*P* < 0.05) than the IPM_CS group, whereas the average proportion of these genera in the CPZ_SAM group was only about 0.3% (Fig. 5E). On day 14, a significantly higher abundance of *Enterococcus* (*P* < 0.01) was observed in the CPZ_SAM group than in the IPM_CS group, and its proportion was more than 80% in the CPZ_SAM group. *Norank_f__Muribaculaceae* and *Bacteroides* were abundant after 14 days of IPM_CS treatment (Fig. 5F). A high abundance of *Eubacterium_fissicatena_group* was observed after 21 and 28 days of IPM_CS treatment (Fig. 5G and H).

### Effect of antibiotic treatment on colonic mucosal tissue and relationship between gut microbiota and cytokines

**Fig 6:**
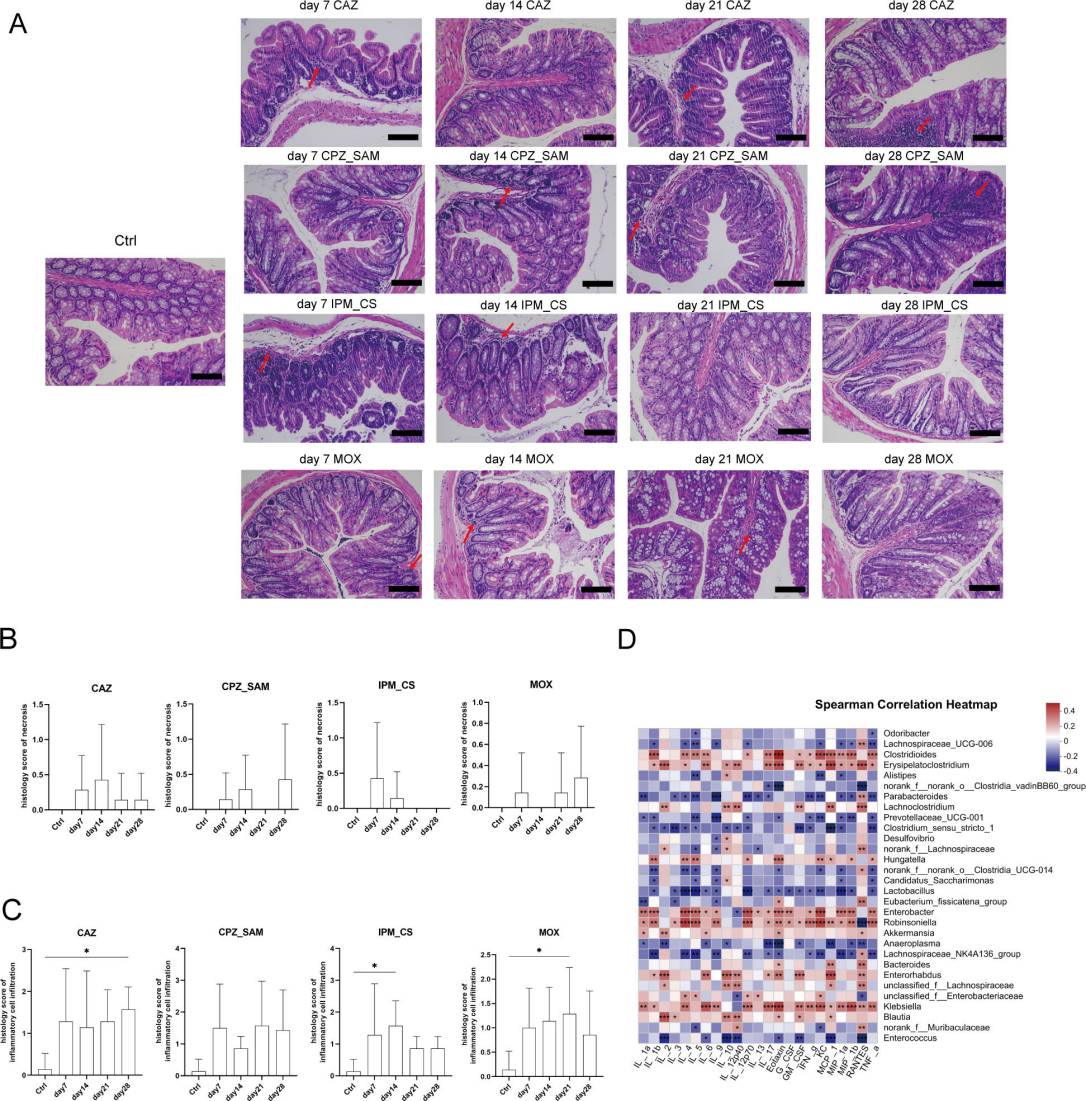
(**A**) H&E staining of colon tissue of mice in the control group and different antibiotic treatment groups. Scale bar: 100 µm. Red arrows indicate inflammatory cell infiltration. (**B**) Pathological scores of necrosis of colon tissue. (**C**) Pathological scores of inflammatory cell infiltration of colon tissue. Data are presented as mean ± SD; *P < 0.05, **P < 0.01, and ***P < 0.001. (**D**) Relationship between microbial community composition (at the genus level) and 23 serum cytokine expression levels. The *R* values are shown in different colors (*R* ≥ 0.1, *P < 0.05, **P < 0.01, and ***P < 0.001). Abbreviations: IPM_CS, imipenem-cilastatin; CPZ_SAM, cefoperazone-sulbactam; CAZ, ceftazidime; MOX, moxifloxacin hydrochloride; Ctrl, control.

Hematoxylin and eosin (H&E) staining of the colon tissue revealed that slight inflammatory cell infiltration was found in the lamina propria and the submucosa after antibiotic treatments (Fig. 6A). The four antibiotic treatments did not cause necrosis with regard to low pathological scores of necrosis (Fig. 6B). Higher pathological scores of inflammatory cell infiltration were observed in four antibiotic groups than those in the Ctrl group (Fig. 6C).

The relationship between gut microbiota composition and inflammatory response was analyzed using Spearman correlation analysis after antibiotic treatment; a correlation heatmap between gut microbiota composition (at the genus level) and 23 serum cytokines was generated (Fig. 6D). The abundance of *Clostridioides*, *Erysipelatoclostridium*, *Enterobacter*, *Robinsoniella*, and *Klebsiella* was positively correlated with the levels of most cytokines (*R* ≥ 0.1, Fig. 6D). In contrast, the abundance of *Lachnospiraceae_NK4A136_group*, *Lactobacillus*, *Clostridium_sensu_stricto_1*, *Prevotellaceae_UCG-001*, and *Parabacteroides* was negatively correlated with the levels of most cytokines (*R* ≥ 0.1, Fig. 6D).

### Changes in serum cytokines after constant antibiotic treatment

**Fig 7:**
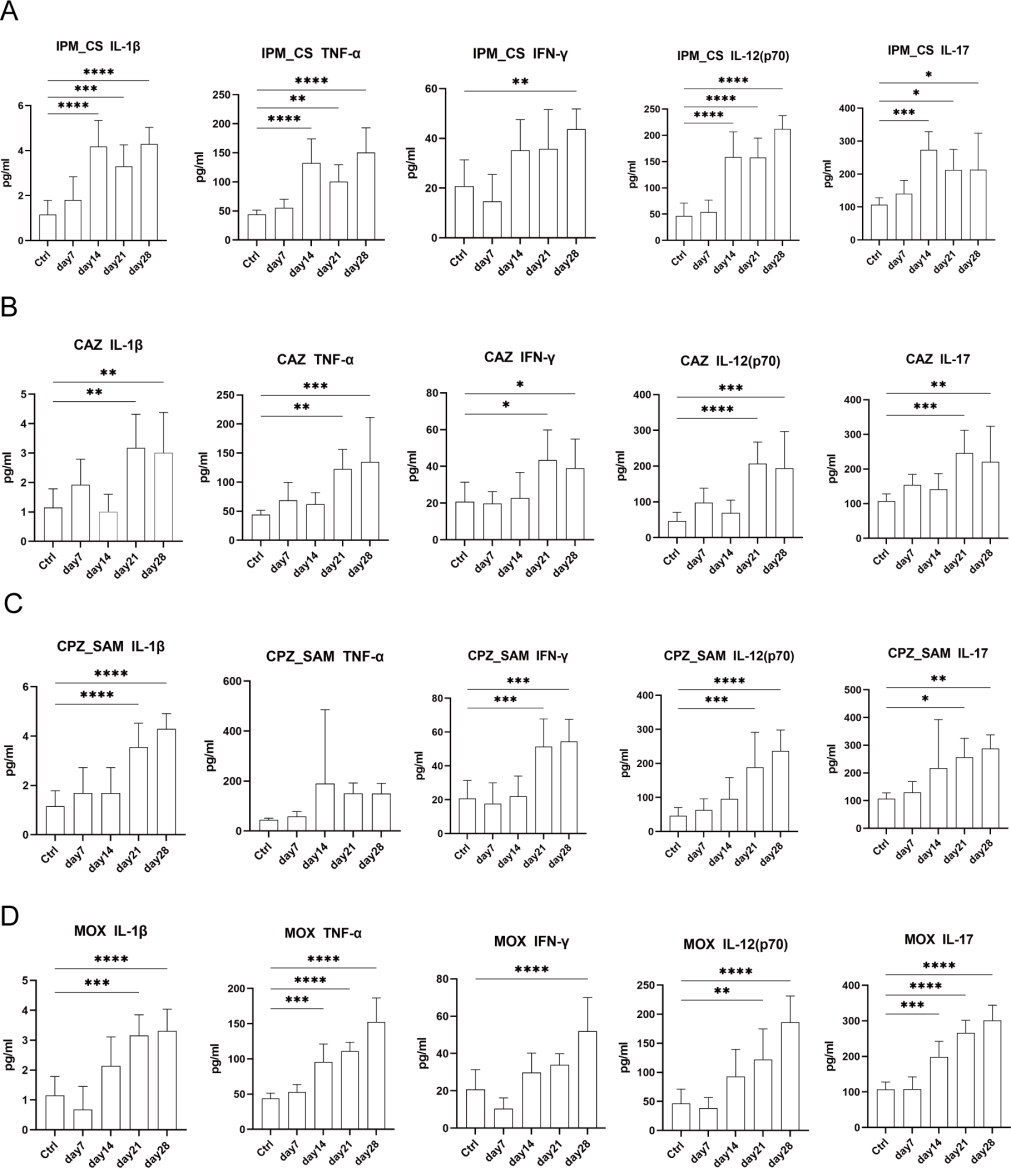
Expression level of some proinflammatory cytokines on days 7, 14, 21, and 28 in antibiotic groups: (**A**) IPM_CS group, (**B**) CAZ group, (**C**) CPZ_SAM group, and (**D**) MOX group. Data were presented as mean ± SD; *P < 0.05, **P < 0.01, and ***P < 0.001. IL-1β, IL-17, and IL-12 (**P70**): interleukins; IFN-γ: interferon-γ; TNF-α: tumor necrosis factor-α; IPM_CS: imipenem-cilastatin; CPZ_SAM: cefoperazone-sulbactam; CAZ: ceftazidime; MOX: moxifloxacin hydrochloride; Ctrl: control.

To investigate the inflammatory response induced by antibiotic treatment in mice, we analyzed the levels of pro-inflammatory cytokines [IFN-γ, TNF-α, IL-1β, IL-17, and IL-12 (p70)] on days 7, 14, 21, and 28 of antibiotic treatment in the four groups (Fig. 7). The levels of IL-1β (*P* < 0.001), TNF-α (*P* < 0.01), IL-12 (p70; *P* < 0.0001), and IL-17 (*P* < 0.05) were significantly higher in IPM_CS group than in the Ctrl group on days 14, 21, and 28 (Fig. 7A). IPM_CS treatment for 14 days was able to cause a significant rise in pro-inflammatory cytokine levels. Compared with the Ctrl group, there was a significant increase in the levels of IL-1β, IFN-γ, IL-12 (p70), and IL-17 in CAZ and CPZ_SAM groups on days 21 and 28 (Fig. 7B and C). On days 21 and 28, the levels of IL-1β (*P* < 0.001), TNF-α (*P* < 0.0001), IL-12 (p70; *P* < 0.01), and IL-17 (*P* < 0.0001) were significantly higher in MOX group than in the Ctrl group (Fig. 7D). The levels of pro-inflammatory cytokines were increasing with time when constantly administrated with CPZ_SAM or MOX (Fig. 7C and D). Additionally, most of the other cytokines were markedly increased on days 21 and 28 in antibiotic treatment groups (Fig. S2 to S5).

## DISCUSSION

Antibiotics have reduced the mortality of infectious diseases and revolutionized the treatment of infectious diseases; however, antibiotics have adverse effects on the gut microbiota and host immune response and health (29). Studies have shown that antibiotic treatment is directly associated with changes in gut microbiota (14, 30). In recent years, the effect of antibiotics on gut microbiota has received widespread attention. However, dynamic changes in gut microbiota caused by continuous antibiotic treatment have not been comprehensively investigated, and few studies have compared and analyzed the gut microbiota composition between two antibiotic treatments. In the present study, the effect of antibiotic treatment on gut microbiota was investigated to provide guidance for antibiotic use from the perspective of gut health. Moreover, the effects of four antibiotic treatments on serum cytokine levels and colon tissue were investigated in this study.

The four antibiotic treatments significantly reduced the species richness and evenness of the gut microbiota and changed the gut microbiota composition and structure; these results are consistent with previous findings (31–33). However, the MOX group had higher alpha diversity on day 7 than on days 14, 21, and 28, which indicated that the MOX treatment for 7 days had a less severe impact on gut microbiota alpha diversity than MOX treatment for other days. Consistent with previously reported findings (31, 32), Firmicutes, Bacteroidota, and Proteobacteria show significant changes in relative abundance during antibiotic treatments in our study. The four antibiotic treatments led to a significant increase in the relative abundance of Firmicutes (*P* < 0.05). In contrast, the four antibiotic treatments caused a significant decrease in the abundance of Bacteroidota. This decrease may increase the likelihood of immune disorders, metabolic syndrome, inflammation, and neurological disorders (34–36). Interestingly, the abundance of Bacteroidota significantly increased in the IPM_CS group on day 14. The majority of healthy gut microbiota is mainly composed of two dominant bacterial phyla—the gram-positive Firmicutes and the gram-negative Bacteroidetes (37). Based on our study findings, the increased abundance of Firmicutes and decreased abundance of Bacteroidetes indicate a greater impact of these four antibiotics on gram-negative bacteria than on gram-positive bacteria. Additionally, the relative abundance of Proteobacteria significantly increased in the CAZ and MOX groups and slightly increased in the IPM_CS and CPZ_SAM groups. Gut inflammation or antibiotic treatment may increase epithelial oxygenation in the colon which may have led to the expansion of Proteobacteria (38). Thus, the increased abundance of Proteobacteria may be a potential diagnostic feature for ecological disorders and the risk of diseases (39).

At the genus level, treatment with antibiotics was found to promote the growth of opportunistic pathogenic bacteria while reducing the abundance of beneficial bacteria. *Enterococcus*, known to be an opportunistic pathogen in the human gut, showed a marked increase in abundance in all antibiotic treatment groups. This increase may be attributed to the antibiotic resistance of *Enterococcus* and the emergence of drug-resistant strains (40). In antibiotic-treated inpatients, *Enterococcus* may cause serious infections including urinary tract infections, intra-abdominal infections, and endocarditis (41). Remarkably, in the CAZ group, the abundance of *Enterococcus* was significantly lower on day 28 than on days 7, 14, and 21 of treatment. In contrast, a rapid, significant increase in the abundance of *Blautia* and *Akkermansia* was observed in the CAZ group on day 28. We speculate that the high abundance of *Blautia* and *Akkermansia* (beneficial bacteria) inhibited the growth of *Enterococcus* (opportunistic pathogenic bacteria) on day 28 in the CAZ group. *Blautia* is a key producer of short-chain fatty acids (SCFAs) and has significant benefits regarding weight loss and anti-inflammation (42). *Akkermansia* has been found to regulate metabolism and immune function and acts as an inhibitor of pathogenic bacteria (43, 44). Our findings were in accordance with a recent study indicating that the abundance of *Akkermansia* increased significantly after long-term antibiotic treatment (45).

In the MOX group, an extremely high abundance of *Enterococcus* (*P* < 0.001) was observed on day 14 compared to its low abundance at other timepoints. Moreover, on day 21, the MOX group showed a significant increase in the abundance of *Klebsiella* (*P* < 0.001), which could cause a range of infectious diseases (46). The high abundance of *Klebsiella* may be related to its resistance to this antibiotic. Therefore, 7-day MOX treatment may have a less severe impact on gut microbiota health than 14-day or 21-day MOX treatment. Both CAZ and MOX are effective against infections caused by non-ESBL-producing gram-negative bacteria. However, the CAZ group showed a higher abundance of *Enterococcus* and lower abundance of *Lactobacillus* on day 7 than the MOX group. The abundance of *Enterobacter* was significantly higher in the CAZ group than in the MOX group on day 14. *Enterobacter* has been reported as an opportunistic pathogen in plants, animals, and humans (47). Thus, MOX treatment had a less severe negative impact on gut microbiota health than CAZ treatment during a 14-day treatment period.

In the IPM_CS group, *Enterococcus* was less abundant on day 14 than on other days, which may be related to the high abundance of *Bacteroides* on day 14. *Bacteroides* species have been recognized as promising probiotics for the development of novel treatments for a range of conditions such as intestinal colitis, immune dysfunction, and metabolic disorders (48). The abundance of *norank_f__Muribaculaceae* on day 14 was the highest in the IPM_CS treatment group. Therefore, treatment with IPM_CS was found to suppress the growth of opportunistic pathogenic bacteria while promoting the abundance of beneficial bacteria on day 14. Gut microbiota induced by IPM_CS treatment might produce less adverse effects on human health on day 14. Additionally, both IPM_CS and CPZ_SAM are effective against ESBL-producing gram-negative bacterial infections. The average proportion of bacteria with significant differences was low between CPZ_SAM and IPM_CS groups on day 7. On day 14, a lower abundance of *Enterococcus* and higher abundance of *Bacteroides* and *norank_f__Muribaculaceae* were observed in the IPM_CS group than in the CPZ_SAM group. Therefore, in terms of the 14-day treatment, IPM_CS may have a less negative impact on gut microbiota health than CPZ_SAM.

Previous research has shown that members of *Lachnospiraceae* and *Muribaculaceae* are major utilizers of mucin monosaccharides and are effective in inhibiting the growth and colonization of *Clostridium difficile* (49). *Lachnospiraceae*, an important family in the gut microbiota, comprise the primary producers of SCFAs (50), which play a role in protecting the gut mucosal barrier and have important immune regulatory functions (51, 52). However, prolonged treatment with the four antibiotics/antibiotic combinations significantly reduced the abundance of these beneficial bacteria, and this effect was long-lasting. Similar findings of changes in gut microbiota have also been reported after treatment with other antibiotics, namely piperacillin-tazobactam, ceftriaxone, tigecycline, and levofloxacin (32). In the present study, we did not further investigate the causes of changes in gut microbiota composition such as high abundance of *Blautia* and *Akkermansia* in the CAZ group on day 28. Additionally, the effects of the four antibiotic treatments on human gut microbiota need to be further studied in clinical settings.

In the colonic tissue, continuous treatment with the four antibiotics resulted in slight inflammatory cell infiltration, and no obvious gut damage was induced by the four antibiotic treatments. Thus, the four treatments did not cause apparent local inflammatory response in the gut. The symbiotic link between gut microbiota and immune system promotes intestinal homeostasis and reduces inflammation (53). Dysbiosis can cause metabolic dysfunction, intestinal barrier damage, and immune disorder, all of which lead to an overproduction of pro-inflammatory cytokines, which promotes inflammation responses (19). Nan et al. found that the levels of inflammatory cytokines were increased after 8- or 28-week antibiotic treatment (45). In our study, 7-day antibiotic treatment did not significantly alter the levels of serum cytokines. However, IPM_CS treatment for 14 days significantly increased the levels of pro-inflammatory cytokines. The levels of most pro-inflammatory cytokines were markedly increased on days 21 and 28 in other antibiotic treatment groups. Additionally, the majority of other cytokines also showed significant increases on days 21 and 28 in the antibiotic treatment groups. These results showed that the inflammatory response might be induced after 21 days of treatment with those antibiotics. We also explored the relationships between gut microbial community composition and serum cytokine levels. Dysbiosis is characterized by an imbalance in gut microbiota, with an increase in pro-inflammatory bacteria and a decrease in anti-inflammatory bacteria, which can lead to inflammatory responses (19). Previously, Zhang et al. demonstrated that antibiotic treatment changed gut microbiota composition, which indirectly triggered an inflammatory reaction (45); the results of the present study support these findings. In our study, *Klebsiella* and *Enterobacter*, which belong to the *Enterobacteriaceae* family, showed a positive correlation with most pro-inflammatory cytokines. *Enterobacteriaceae* play a crucial role in producing lipopolysaccharide, which has been demonstrated to induce chronic inflammation through the lipopolysaccharide-binding protein signaling pathway (54). SCFAs exhibit immunomodulatory and anti-inflammatory properties and thus may help to prevent excessive inflammatory responses (55, 56). The increased level of serum cytokines was related to a significant decrease in short-chain fatty acid-producing bacteria such as *Lachnospiraceae_NK4A136_group and Prevotellaceae_UCG-001*.

In summary, antibiotic treatment had a significant impact on the gut microbiota composition. Treatment with four antibiotics/antibiotic combinations increased the abundance of opportunistic pathogens and lowered the abundance of beneficial bacteria. The results also suggested that 21 days of antibiotic treatments may lead to the activation of systemic inflammatory response; however, the four antibiotic treatments did not significantly induce a local inflammatory response in the gut. These findings highlighted the potential risk associated with the use of these antibiotics. Therefore, it is important to use antibiotics with caution and prudence. Additionally, in terms of effects on gut microbiota health, MOX is recommended for short-term use. The effect of MOX treatment on gut microbial health may be less severe compared to CAZ treatment during the 14-day antibiotic treatment. Imipenem-cilastatin may have a less severe impact on gut microbiota health than cefoperazone-sulbactam during the 14-day antibiotic treatment. However, in the present study, we did not further investigate the causes of changes in gut microbiota composition such as high abundance of *Blautia* and *Akkermansia* in the CAZ group on day 28. The effects of the four antibiotic treatments on human gut microbiota need to be further studied in clinical settings.

## MATERIALS AND METHODS

### Experimental design

Male BALB/c mice (6 weeks old) were procured from Shanghai SLAC Laboratory Animal Co. Ltd. To minimize the effect of environmental factors on the experimental results, the mice were allowed to acclimate for 2 weeks and were provided with sufficient nutrition in a rigorously maintained pathogen-free environment. At the end of the acclimation period, 140 male BALB/c mice were randomly divided into the following groups: normal saline (Ctrl), IPM_CS, CAZ, CPZ_SAM, and MOX. The dose administered to mice was calculated using the following formula based on drug doses administered to humans: dose (mg/kg) administered to mice = dose (mg/kg) administered to adult humans × 60 kg × 0.0030/0.02 kg. The medications were administered via subcutaneous injection daily for up to 4 weeks at the recommended dosages (in mg per 20 g mice body weight): 3 (IPM_CS), 3 (CAZ), 6.01 (CPZ_SAM), and 1.2 (MOX).

Mice were weighed on days 0, 3, 7, 14, 21, and 28. The fecal appearance of mice was recorded for 28 days. On days 7, 14, 21, and 28 of continuous antibiotic therapy, seven mice were sacrificed at each timepoint in the same antibiotic treatment group in order to collect serum, feces, and colon tissues. For example, BALB/c mice in CAZ7, CAZ14, CAZ21, and CAZ28 groups (*n* = 7) were sacrificed on days 7, 14, 21, and 28 of CAZ treatment, respectively, in order to collect serum, feces, and colon tissues. These samples were immediately frozen and stored at −80°C until analysis. The experimental design scheme is shown in Fig. 1A.

### 16S rRNA gene sequence analysis

Bacterial DNA was extracted from frozen fecal samples using the QIAamp DNA Stool Mini Kit (Qiagen, Hilden, Germany) according to the manufacturer’s instructions. The V3–V4 variable regions of bacterial 16S rRNA genes were amplified using the upstream primer 338F (5′-ACTCCTACGGGAGGCAGCAG-3′) and the downstream primer 806R (5′-GGACTACHVGGGTWTCTAAT-3′) (57) Sequencing was performed on the Illumina Miseq pE300/NovaSeq pE250 platform. The Ribosomal Database Project (RDP) classifier (58) (http://sourceforge.net/projects/rdp-classifier/) and the SILVA 16S rRNA gene database (v138) were used to annotate Operational Taxonomic Unit (OTU) taxonomy with a 70% confidence threshold and to determine the community composition of each sample at different taxonomic levels. Alpha diversity is a comprehensive indicator signifying species richness and evenness. 16S functional prediction analyses were performed using PICRUSt2 software (59) (version 2.2.0). Mothur software (60) (http://www.mothur.org/wiki/Calculators) was used to calculate the Chao index. Beta diversity signifies species variability among different microbial communities. The similarity in microbial community structure between samples was examined using PCoA based on weighted UniFrac distance. Species with significant differences in microbial communities between multiple groups were analyzed using the Kruskal-Wallis rank sum test.

### Quantification of serum cytokines

Serum samples of mice in each group were collected in four batches on days 7, 14, 21, and 28. Serum cytokine levels were analyzed using the Bio-plex pro Mouse Cytokine 23-plex panel (Bio-Rad). The following 23 serum cytokines were determined: interleukin-1α (IL-1α), IL-1β, IL-2, IL-3, IL-4, IL-5, IL-6, IL-9, IL-10, IL-12 (p40), IL-12 (p70), IL-13, IL-17, interferon gamma (IFN-γ), eotaxin, granulocyte colony-stimulating factor, granulocyte-macrophage colony-stimulating factor, monocyte chemoattractant protein (MCP)−1, macrophage inflammatory protein 1α (MIP-1α), MIP-1β, regulated upon activation, normal T cell expressed and secreted factor (RANTES), and tumor necrosis factor α (TNF-α).

### Histological assessment of colon tissues

Colon tissue was fixed for 24 h in a 10% formalin solution and then was dehydrated and embedded in wax. The embedded tissues were cut into ultra-thin sections using a microtome (RM2016, Leica Instruments Shanghai Ltd.) and stained with H&E.

The histological score was determined by observing H&E-stained colon sections under a microscope (NIKON ECLIPSE E100) and scored on the basis of pathological changes in the colon tissue according to the scoring methods proposed by Peter Mann et al. The score was based on necrosis or inflammatory cell infiltration, with a range of 0–4 (0 indicating normal, 1 indicating very slight lesions, 2 indicating slight lesions, 3 indicating moderate lesions, and 4 indicating severe lesions).

### Statistical analysis

Statistical analysis was performed using the Student’s *t* test for normally distributed data and the Wilcoxon test for non-normally distributed data. One-way analysis of variance was used for analyzing normally distributed data, whereas the Kruskal-Wallis H-test was used for non-normally distributed data. Both tests are suitable for analyzing data consisting of three or more groups. The non-parametric Spearman correlation test was used to investigate the relationship between microbial community composition (at the genus level) and serum cytokine expression levels. Statistical analysis was conducted using GraphPad Prism (version 9.0) and R software. Data were presented as mean ± SEM.

## Data Availability

The sequence data were deposited in the NCBI BioProject (PRJNA1081792).

## SUPPLEMENTAL MATERIAL

Supplemental material spectrum.03095-23-s0001.docx Table S1; Fig. S1 to S5. Record of fecal traits; comparison of Shannon index between the control group and different antibiotic treatment groups; expression level of other inflammatory cytokines.

ASM does not own the copyrights to Supplemental Material that may be linked to, or accessed through, an article. The authors have granted ASM a non-exclusive, world-wide license to publish the Supplemental Material files. Please contact the corresponding author directly for reuse.
