# Supplementary material for: Alterations in gut microbiota and inflammatory cytokines after administration of antibiotics in mice
Source: Microbiol Spectr. 2024 Jun 20;12(8):e03095-23. doi: 10.1128/spectrum.03095-23 (PMC11302321; doi:10.1128/spectrum.03095-23)
Supplement: Supplemental material — Table S1; Fig. S1 to S5. Record of fecal traits; comparison of Shannon index between the control group and different antibiotic treatment groups; expression level of other inflammatory cytokines. [file spectrum.03095-23-s0001.docx]

Supplementary materials

**Table S1.** Record of fecal traits

| **Time/day** | **CAZ** | **CPZ_SAM** | **IPM_CS** | **MOX** | **Ctrl** |
| --- | --- | --- | --- | --- | --- |
| 0 | normal | normal | normal | normal | normal |
| 1 | normal | normal | normal | normal | normal |
| 2 | normal | normal | normal | normal | normal |
| 3 | normal | normal | normal | normal | normal |
| 4 | normal | normal | normal | normal | normal |
| 5 | normal | soft | normal | normal | normal |
| 6 | normal | soft | normal | soft | normal |
| 7 | soft | soft | soft | soft | normal |
| 8 | soft | soft | soft | soft | normal |
| 9 | soft | soft | soft | soft | normal |
| 10 | soft | soft | soft | soft | normal |
| 11 | soft | soft | soft | soft | normal |
| 12 | soft | soft | soft | soft | normal |
| 13 | soft | soft | soft | soft | normal |
| 14 | soft | soft | soft | soft | normal |
| 15 | soft | soft | soft | soft | normal |
| 16 | soft | soft | soft | soft | normal |
| 17 | soft | normal | soft | soft | normal |
| 18 | soft | normal | soft | normal | normal |
| 19 | soft | normal | soft | normal | normal |
| 20 | soft | normal | soft | normal | normal |
| 21 | normal | normal | normal | normal | normal |
| 22 | normal | normal | normal | normal | normal |
| 23 | normal | normal | normal | normal | normal |
| 24 | normal | normal | normal | normal | normal |
| 25 | normal | normal | normal | normal | normal |
| 26 | normal | normal | normal | normal | normal |
| 27 | normal | normal | normal | normal | normal |
| 28 | normal | normal | normal | normal | normal |

**FIG S1** Comparison of Shannon index between the control group and different antibiotic treatment groups, indicating that antibiotic treatment altered the alpha diversity; **p* < 0.05, ***p* < 0.01, and ***(p < 0.001).

**Abbreviations:** IPM_CS: imipenem-cilastatin; CPZ_SAM: cefoperazone–sulbactam; CAZ: ceftazidime; MOX: moxifloxacin hydrochloride; Ctrl: control.

**
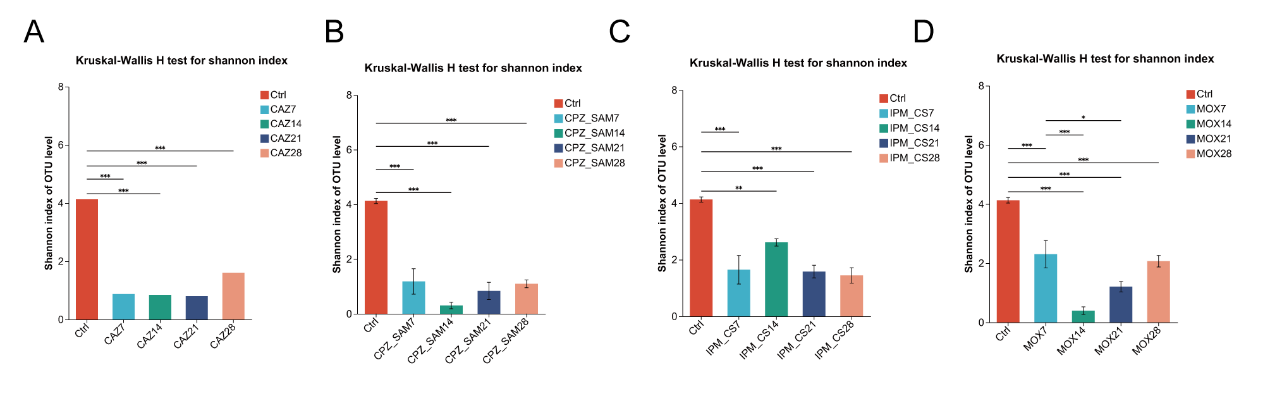
**

**FIG S2** Expression level of other inflammatory cytokines on days 7, 14, 21, and 28 in IPM_CS group. Data were presented as mean ±SD; **p* < 0.05, ***p* < 0.01, ****p* < 0.001.

**Abbreviations:** IL-1α, IL-3, IL-4, IL-5, IL-6, IL-9, IL-10, IL-12 (p40), IL-13 : interleukins; eotaxin; G-CSF: granulocyte colony stimulating factor; GM-CSF: granulocyte–macrophage colony stimulating factor; MCP-1: monocyte chemoattractant protein; MIP-1α, MIP-1β: macrophage inflammatory protein; RANTES: regulated upon activation, normal T cell expressed and secreted factor.

**
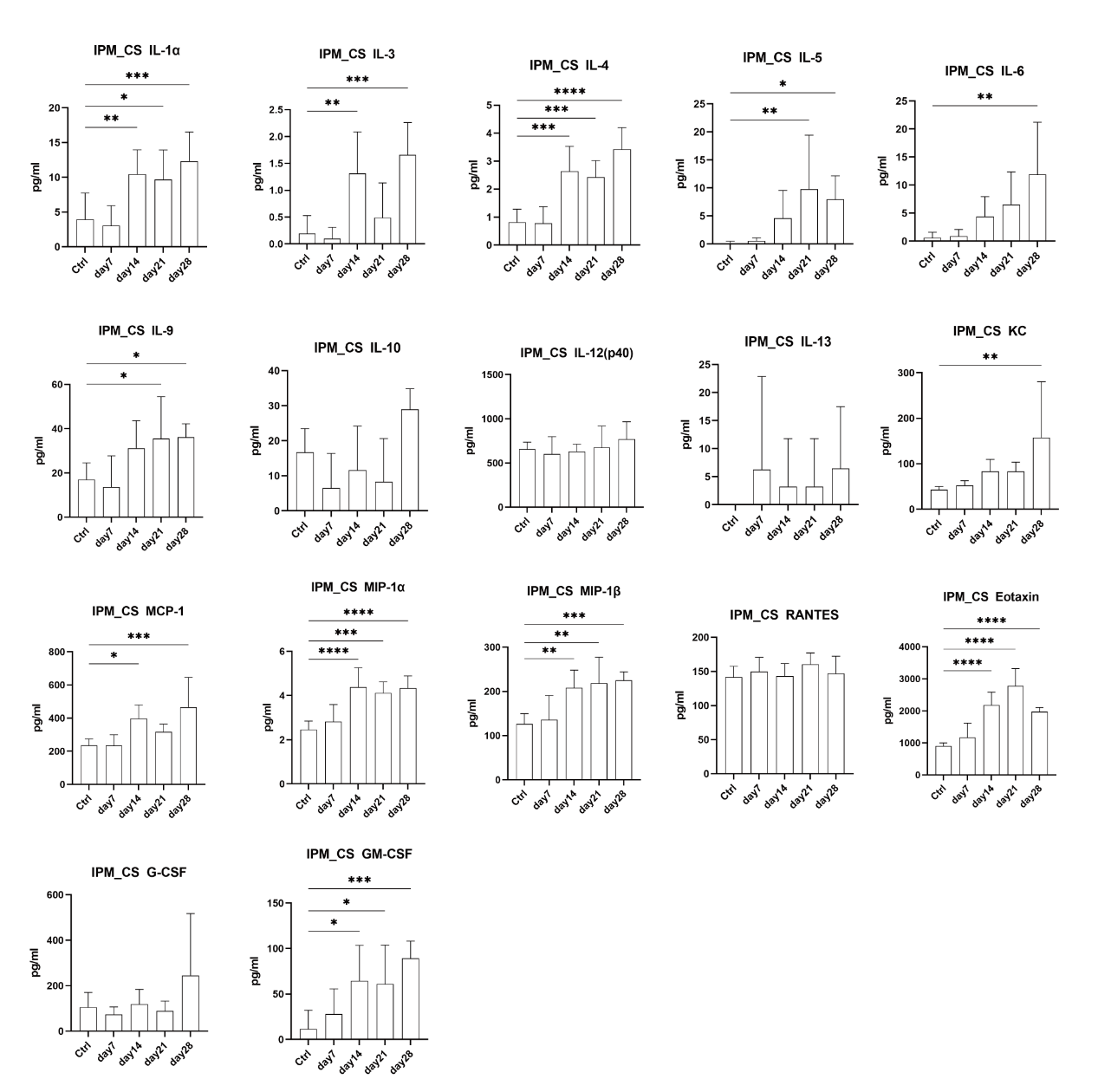
**

**FIG S3** Expression level of other inflammatory cytokines on days 7, 14, 21, and 28 in CAZ group. Data were presented as mean ± SD; **p* < 0.05, ***p* < 0.01, ****p* < 0.001.

**Abbreviations:** IL-1α, IL-3, IL-4, IL-5, IL-6, IL-9, IL-10, IL-12 (p40), IL-13 : interleukins; eotaxin; G-CSF: granulocyte colony stimulating factor; GM-CSF: granulocyte–macrophage colony stimulating factor; MCP-1: monocyte chemoattractant protein; MIP-1α, MIP-1β: macrophage inflammatory protein; RANTES: regulated upon activation, normal T cell expressed and secreted factor.

**
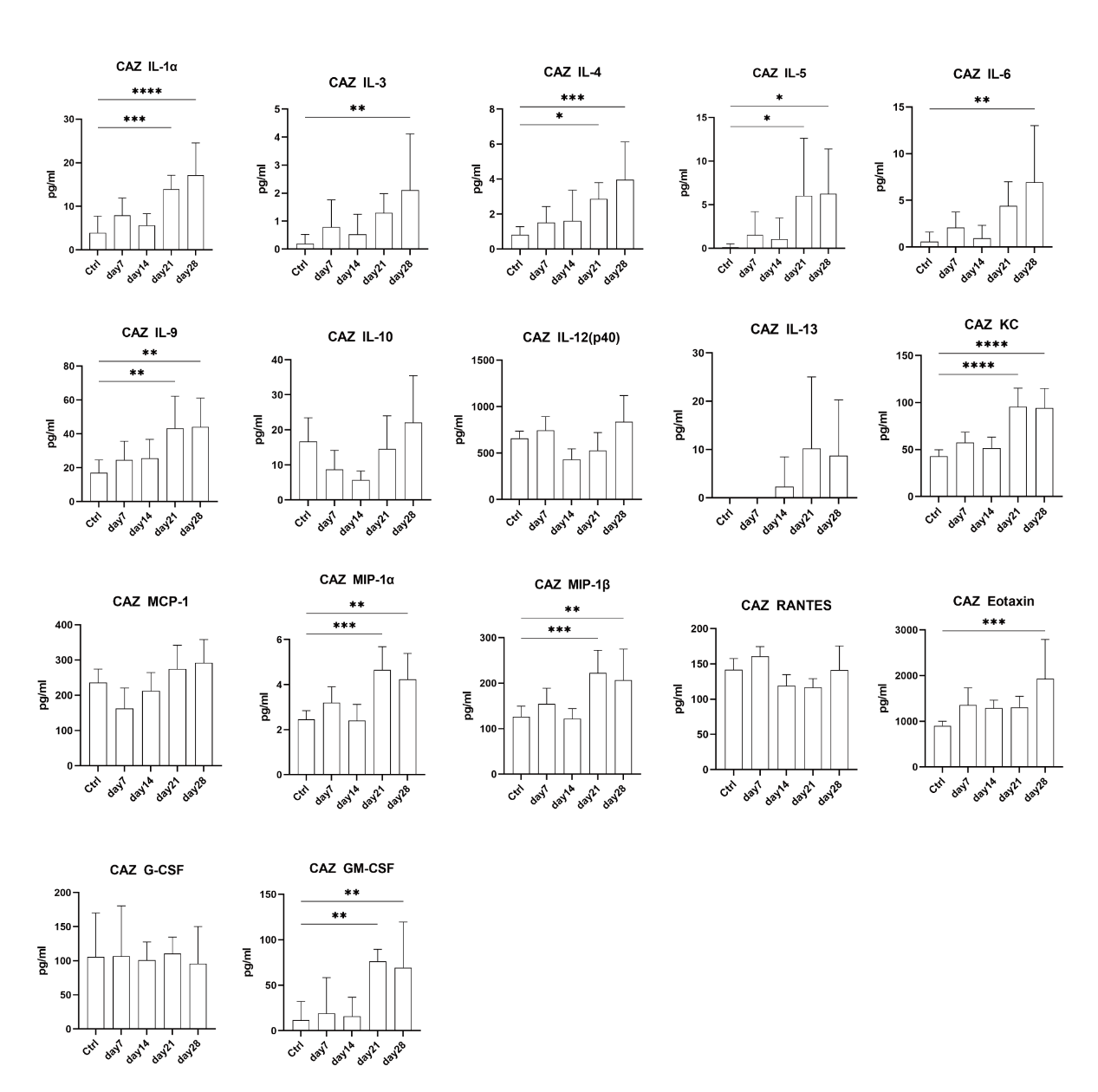
**

**FIG S4** Expression level of other inflammatory cytokines on days 7, 14, 21, and 28 in CPZ_SAM group. Data were presented as mean ± SD; **p* < 0.05, ***p* < 0.01, ****p* < 0.001.

**Abbreviations:** IL-1α, IL-3, IL-4, IL-5, IL-6, IL-9, IL-10, IL-12 (p40), IL-13 : interleukins; eotaxin; G-CSF: granulocyte colony stimulating factor; GM-CSF: granulocyte–macrophage colony stimulating factor; MCP-1: monocyte chemoattractant protein; MIP-1α, MIP-1β: macrophage inflammatory protein; RANTES: regulated upon activation, normal T cell expressed and secreted factor.

**
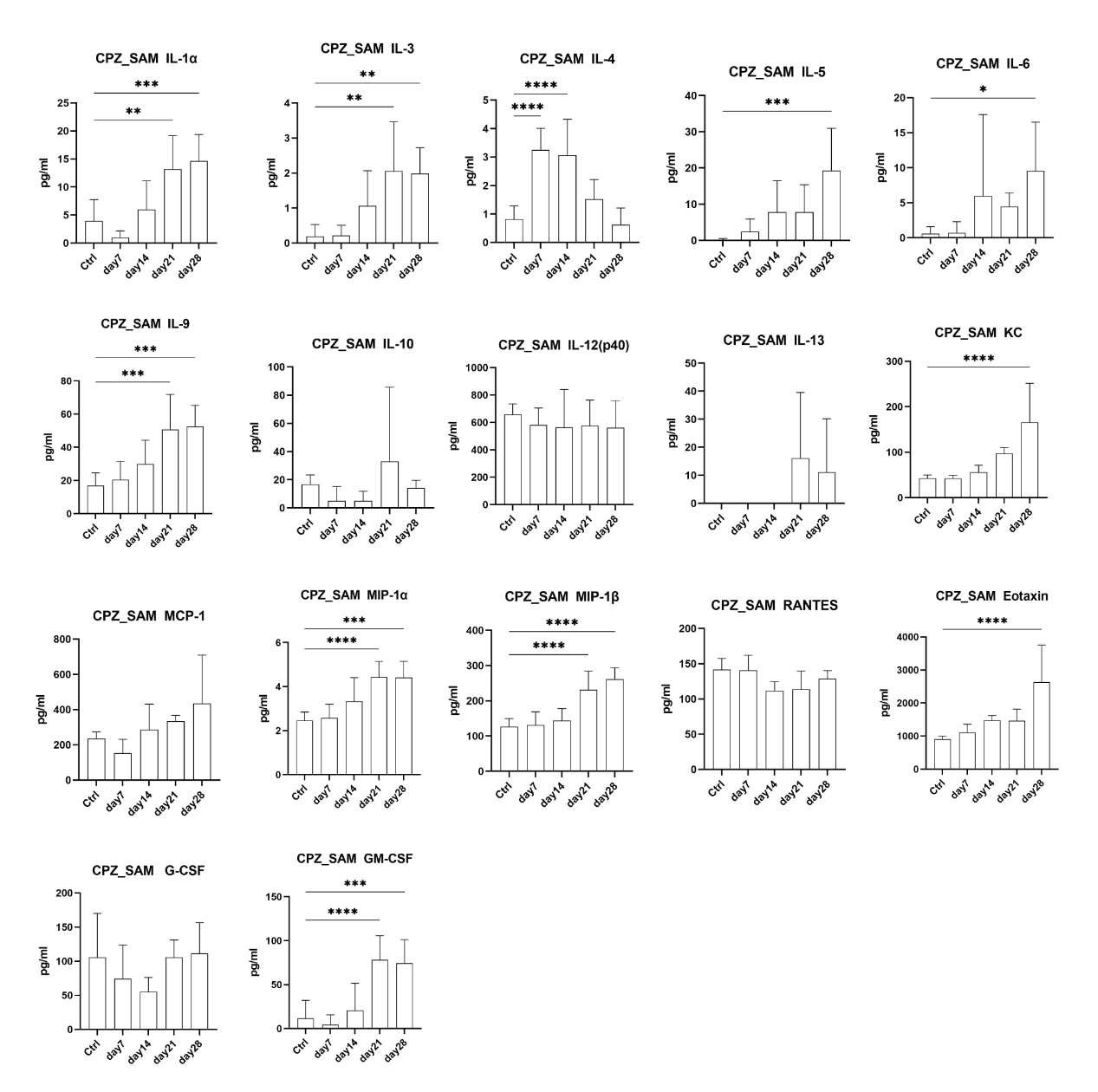
**

**FIG S5** Expression level of other inflammatory cytokines on days 7, 14, 21, and 28 in MOX group. Data were presented as mean ± SD; **p* < 0.05, ***p* < 0.01, ****p* < 0.001.

**Abbreviations:** IL-1α, IL-3, IL-4, IL-5, IL-6, IL-9, IL-10, IL-12 (p40), IL-13 : interleukins; eotaxin; G-CSF: granulocyte colony stimulating factor; GM-CSF: granulocyte–macrophage colony stimulating factor; MCP-1: monocyte chemoattractant protein; MIP-1α, MIP-1β: macrophage inflammatory protein; RANTES: regulated upon activation, normal T cell expressed and secreted factor.

**
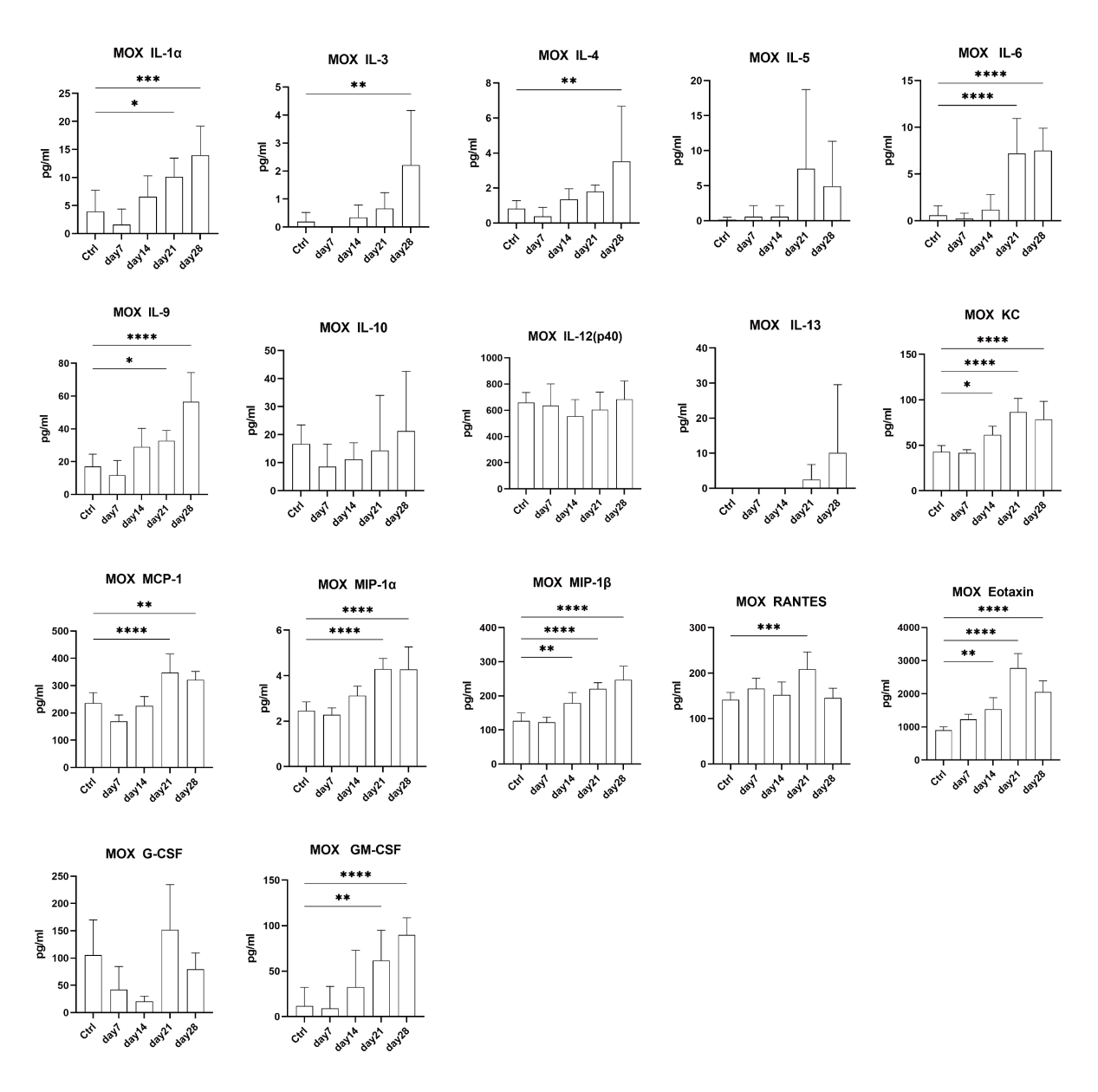
**
